# Accelerating Spatial Cross-Matching on CPU-GPU Hybrid Platform With CUDA and OpenACC

**DOI:** 10.3389/fdata.2020.00014

**Published:** 2020-05-08

**Authors:** Furqan Baig, Chao Gao, Dejun Teng, Jun Kong, Fusheng Wang

**Affiliations:** ^1^Computer Science Department, Stony Brook University, Stony Brook, NY, United States; ^2^Computer Science Department, New York University, New York, NY, United States; ^3^Mathematics and Statistics Department, Gerogia State University, Atlanta, Georgia; ^4^Biomedical Informatics Department, Stony Brook University, Stony Brook, NY, United States

**Keywords:** spatial-cross-matching, spatial-join, gpu, gpgpu, cpugpu-hybrid, geospatial, openacc

## Abstract

Spatial cross-matching operation over geospatial polygonal datasets is a highly compute-intensive yet an essential task to a wide array of real-world applications. At the same time, modern computing systems are typically equipped with multiple processing units capable of task parallelization and optimization at various levels. This mandates for the exploration of novel strategies in the geospatial domain focusing on efficient utilization of computing resources, such as CPUs and GPUs. In this paper, we present a CPU-GPU hybrid platform to accelerate the cross-matching operation of geospatial datasets. We propose a pipeline of geospatial subtasks that are dynamically scheduled to be executed on either CPU or GPU. To accommodate geospatial datasets processing on GPU using pixelization approach, we convert the floating point-valued vertices into integer-valued vertices with an adaptive scaling factor as a function of the area of minimum bounding box. We present a comparative analysis of GPU enabled cross-matching algorithm implementation in CUDA and OpenACC accelerated C++. We test our implementations over Natural Earth Data and our results indicate that although CUDA based implementations provide better performance, OpenACC accelerated implementations are more portable and extendable while still providing considerable performance gain as compared to CPU. We also investigate the effects of input data size on the IO / computation ratio and note that a larger dataset compensates for IO overheads associated with GPU computations. Finally, we demonstrate that an efficient cross-matching comparison can be achieved with a cost-effective GPU.

## 1. Introduction

Spatial data generation and availability have exploded over recent years due to the proliferation of GPS devices, location-based services, high-resolution imaging technologies and volunteered geographic information (Simion et al., 2012) systems, etc. For instance, as of February 2020, OpenStreetMap (OSM), one of the most used collaborative geographic data platform, has gathered about 5.75 billion data points from more than 6 million registered users performing 3.5 million map changes per day on average. While geospatial analysis has become essential to help guide decision making in industrial as well as scientific domains (Wang et al., 2015), the unprecedented scale and rate of data generation make it increasingly difficult to design effective real-world spatial querying solutions.

Geospatial data analysis encompasses a wide set of real-world applications. For instance, traffic engineering, urban planning, climate modeling, medical image analysis, etc. all typically include combinations or variations of spatial proximity queries between objects, nearest neighbor queries that determine the datasets closest to the query point, spatial cross-matching queries, window-based queries, and queries to discover global spatial pattern, such as finding spatial clusters (Zhang et al., 2003; Aji et al., 2013; Wang et al., 2015; Liang et al., 2016). One important spatial query is the spatial cross-matching operation which is used to compare geospatial objects or data points from multiple surveys. Spatial cross-matching is essential to many real-world use cases, such as finding all pairs of datasets with inter-distance less than a threshold in astronomical catalogs (Jia and Luo, 2016), and evaluating segmentation algorithms in pathological datasets (Wang et al., 2012; Baig et al., 2015).

As massive amounts of data are generated, a gap exists between the efficiency of the analytics system and the amounts of data that need to be processed. Geospatial data processing thus is challenged by both data- and compute- intensive characteristics. Spatial queries are supported by traditional spatial database management systems (SDBMSs), such as PostGIS (Committee, 2018), Oracle Spatial (Oracle, 2018), and ArcGIS (Esri, 2018). These databases support various spatial data types, such as point, line, and polygon, and operations to compute distance, intersection, and containment, etc. However, their capabilities are fairly limited by a lack of effective spatial partitioning mechanism to balance workloads and the difficulty to manage computationally intensive operations, such as geometric computations. Therefore, to mitigate these limitations they typically rely on parallel computing capabilities to increase the efficiency of geospatial data processing.

The emergence of MapReduce enables data partitioning and distribution of partitioned data onto multiple workers and conduct spatial queries in parallel on commodity hardware (Aji et al., 2014; Eldawy, 2014; Vo et al., 2014). This greatly increases the efficiency of the processing system to deal with large spatial query workloads. Recently, the rapid availability of low cost but powerful high memory systems has introduced iterative in-memory processing models in a distributed environment. Coupled with spatial processing engines, systems (such as Jia Yu, 2015; You and Zhang, 2015; Xie et al., 2016; Baig et al., 2017) further increase the efficiency and performance of scalable distributed spatial data processing. However, while data partitioning is effective in improving the query throughput, it is not intended to address extensive geometric computation.

The compute-intensive challenge is well-suited to be handled by *General Purpose Graphical Processing Unit* (GPGPU). Recent works have proposed to utilize GPU in order to enable high-performance spatial queries for computationally intensive operations, such as spatial cross-matching or overlay of spatial objects from images or maps. The GPU provides unprecedented computing power by exploiting extreme parallelism which can achieve up to 3× speedup compared to a multi-core CPU with reasonable porting effort (Prasad et al., 2015). The most fundamental characteristic of applications suitable for GPU is being embarrassingly parallel where tasks function independently. Geospatial data processing that directly falls into this category includes points in polygon test, forming k clusters in *Geographic Information System* (GIS) grid, and computation of raster images. In general geospatial problems based on regular grids/pixels are well-suited to be executed in parallel on GPU. However, contemporary algorithms for geospatial operations, such as computing intersection and union of polygon pairs cannot naively be parallelized and require extra efforts to be re-design according to GPU architecture. For instance, PixelBox algorithm transforms the vector-based geometry representation into raster representation, then computes the intersection and union area of polygonal pairs (Wang et al., 2012).

Despite being able to address the computational aspect of spatial processing, GPU is not by any means a panacea and does have its own limitations. These include but are not limited to data movement cost, lower clock speed, slower memory access, etc. Additionally, modern CPUs are also able to utilize parallelism to some extent. Concentrating a particular job on either of the two resources only would leave the overall system highly underutilized. Furthermore, specifically in terms of spatial processing, different stages in the query pipeline have independent characteristics and dependencies making some of them suitable for CPU while others more fitting to be executed on GPU.

Inspired by these observations, our work intends to explore a hybrid approach by embracing heterogeneous computing strategy including both CPU and GPU. This can not only increase the overall resource utilization but also multiply the throughput of task processing by using multiple CPU threads along with GPU. In a hybrid platform, an execution engine determines the device (CPU/GPU) to run the tasks based on factors, such as potential speedup gain and data movement cost (Aji et al., 2014). A major challenge of this approach is to determine an efficient mechanism to schedule the tasks on CPU and/or GPU while ensuring that all devices are fully utilized. When a GPU is selected to accelerate data processing, the price of GPU is another factor that needs to be taken into consideration. GPUs with different prices can be used in applications corresponding to various use cases and speedup requirements.

In addition to providing generic GPU algorithms for spatial cross-matching, in this study we also evaluate various implementations of the proposed algorithm on CPU and GPU in CUDA and OpenACC (Nvidia, 2019). Programming language and interface play an important role in the research and development life cycle of an algorithm. Algorithms implemented in well-known generic languages usually tend to be more portable, easier to maintain and extend. While CUDA, a specialized parallel computing application programming interface, has been the de facto (Nvidia) GPU programming standard for over a decade, OpenACC has emerged as more suitable for hybrid platforms over the recent years. Unlike CUDA, OpenACC is a directive-based parallel computing model developed by NVidia and its partners to simplify programming of heterogeneous hardware platforms equipped with multiple processing units, such as CPUs and GPUs. Developers can implement their algorithms in well-known languages, such as C/C++. The same code can then be annotated with OpenACC compiler directives to be accelerated on GPUs.

Specifically in this study, we identify, explore and evaluate spatial cross-matching subtasks suitable for acceleration on our proposed hybrid platform. We implemented several extensions and variations of PixelBox algorithm to perform these subtasks efficiently on CPU and GPU. In particular, for GPU, we investigate different parameters and trade-offs for CUDA based implementation vs. OpenACC accelerated code. We extend our previous work (Wang et al., 2012; Aji et al., 2014; Gao et al., 2018) from the user case of pathological imaging analysis to enable spatial join query processing of geospatial datasets. There are several differences between the two types of applications: the pathology image data considers each polygon with edges along vertical/horizontal directions and with vertices in integer-based coordinates. However, in general, geospatial polygons can have edges with any orientation and their vertices can be represented by floating point numbers. To accommodate such geospatial datasets to GPU, we propose an adaptive scaling strategy as a function of the area of minimum bounding box of query polygons.

This study is based on the following contributions from our previous work (Gao et al., 2018).

- We proposed and studied a hybrid CPU-GPU architecture to accelerate spatial cross-matching queries on a single node.
- We employed an adaptive scaling factor method to convert geospatial datasets from floating point-valued vertices to integer-valued vertices to accommodate GPU computation with pixelization method.
- We investigated the effect of input data size on proposed system performance including IO and computation time.
- We studied the effect of two different GPUs on the speedup ratio to process geospatial datasets and demonstrate the feasibility of computation with a cost-effective GPU.

Extending on above, our main contributions in this study are summarized as follows

- We study the trade-off between performance and portability for GPU accelerated code
- We implement CPU-GPU hybrid pipeline in C++ accelerated by OpenACC
- We implement Pixel based GPU cross-matching algorithm in CUDA
- We evaluate performance of pixel based algorithm vs. geometry based algorithm implemented on CPU
- We analyze performance of CUDA vs. OpenACC for pixel based algorithm on GPU

The rest of the paper is organized as follows. We first present necessary background and related research in section 2. Section 3 overviews the architectural components of the CPU-GPU platform along with the updates to the underlying engine. For the sake of completeness, section 4 highlights spatial cross-matching workflow identified in our previous work (Gao et al., 2018) whereas section 5 explains the details of multiple adaptations of geometry and pixel based spatial cross-matching tasks specifically implemented for CPU, GPU and Hybrid processing units. Experimental evaluation with analysis of these implementations on real world dataset is presented in section 6 which is followed by conclusion.

## 2. Background and Related Work

### 2.1. Spatial Cross Matching

Spatial cross-matching defines the set of operations used to cross-compare two or more datasets with spatial predicates. In spatial literature, the operation can be characterized as an extension of spatial join. Spatial cross-matching essentially is a spatial join operation followed by computation of spatial features, such as area of intersection and union area etc. of query spatial objects.

The datasets for spatial join usually cover a wide range of spatial data types. For example, combining *Point-Point* dataset produces *Multipoint* result, *LineString-Polygon* results in *Multi-LineString, Point-Polygon* data produces *Multi-Point* etc. However, this study is mainly focused on *Polygon-Polygon* datasets.

The cross-matching query itself relies on several predicates to work with. For instance, *intersects*, tests if spatial objects in datasets intersect with each other, *contains* tests if one object is fully inside another, etc. These predicates generally translate directly to Boolean operators from the domain of *polygon overlay*. For instance, *AND* corresponds to *intersects* and *OR* directly translates to *union* predicates.

Based on these predicates, further computations are performed on these spatial objects (mainly polygons in this study), such as their intersection area, union area, and their respective ratios. The results from these computations are then used to define functions, such as the Jaccard similarity coefficient etc. to perform complex spatial analytics.

### 2.2. Object Level Parallelism

Spatial cross-matching is a highly compute-intensive operation. In particular, Traditional Spatial Database Management Systems (SDBMS) have major limitations on managing and querying large scale spatial data. A performance profiling of a typical spatial cross-matching operation on PostGIS (Committee, 2018), a popular open-source SDBMS, reveals an overwhelming 90% of the total query execution time was spent in computing spatial cross-matching predicates (Wang et al., 2012). Additionally, SDBMSs also suffer from the high overhead of data loading as another major bottleneck (Özsu and Valduriez, 2011; Aji et al., 2013).

SDBMSs (Adler, 2001; Committee, 2018; IBM, 2018) can rely on parallel DBMS architectures to partition data on multiple disks. This not only allows for parallel query execution but also tends to reduce the overall I/O bottleneck. However, the general-purpose spatial algorithms used by underlying libraries for SDBMSs are typically not designed for parallel execution (Wang et al., 2012). In addition to that, data skew, introduced by partitioning, adversely affects parallel query performance.

Map-Reduce based distributed spatial processing systems (Nishimura et al., 2011; Eldawy, 2014; Jia Yu, 2015; You and Zhang, 2015; You et al., 2015; Xie et al., 2016; Baig et al., 2017) are able to scale out and efficiently distribute geospatial queries. Furthermore, in-memory frameworks built on Apache Spark, etc., can take advantage of iterative processing model to further mitigate IO overheads. However, the underlying processing engines of these systems handle each object independently and thus can at most parallelize at object level only. While the overall system performance is commendable in terms of scalability and parallelism, geometric computation still accounts for the majority of query cost. For example, despite utilizing distributed parallelism and iteratively processing geospatial data in memory, a cross-matching query in SparkGIS (Baig et al., 2017) was dominated by spatial computations.

### 2.3. GPU Enabled Intra-Object Level Parallelism

Recent research in the geospatial paradigm has proposed algorithms that exploit intra-object parallelism using high throughput Graphical Processing Units. Puri et al. (2015) studied traditional CPU and GPU based geometry computation platforms and concluded that CPU based traditional algorithms are not suited for performance gain when executed on GPU. They proposed to develop new spatial computation algorithms optimized for GPU architecture. Specifically, they concluded that such algorithms need to accommodate the Single-Instruction Multiple-Threads (SIMT) paradigm of GPU.

Lo et al. (2011) proposed a parallel rectangle intersection algorithm and observed a 30× speedup for 10 million rectangles when executed on GPU. They estimated and assigned at most *T* rectangles to each thread block, and each thread compared one rectangle with all rectangles in the same cell. Similarly, You et al. (2016) proposed and implemented a GPU accelerated polyline intersection algorithm specifically designed for spatial join operation. They assigned a pair of polyline to a GPU thread block and within each block, they used each thread to check a pair of line segments intersection. They were able to accelerate the spatial join operation by 20% as compared to CPU. Using the LDE framework, they further achieved 22.6× speedup on their high-end workstation (Zhang et al., 2015b). Yampaka and Chongstitvatana (2013) implemented overlap and spatial join operations on GPU over rectangles. Zhang and You (2012) developed a GPU-based spatial join framework to conduct the point-in-polygon (PIP) test using both the block and thread-level parallelisms.

In one of our previous works, we proposed the PixelBox algorithm for speeding up cross-comparison queries for pathology images (Wang et al., 2012). This approach first partitions the Minimum Bounding Box (MBB) of each polygon pair into boxes at coarser granularity and determines the relative position of each box with respect to the polygon pair, then for boxes with a small number of pixels, it reduces the computation into the pixel-in-polygon test that is well-suited for GPU. It achieved 18× speedup compared with a parallelized spatial database approach. Furthermore, we incorporated the PixelBox algorithm into a MapReduce framework (Aji et al., 2014).

Despite being able to achieve high intra-object level parallelism, all of the above-mentioned strategies are GPU centric and thus have to adapt all workloads to suit GPU inherent patterns, such as lower clock speeds, memory access, etc. Contrarily, our proposed model takes a hybrid approach to schedule workloads on CPUs as well as GPUs simultaneously thus providing higher resource utilization and consequently better support and performance.

## 3. CPU-GPU Hybrid Spatial Query System Overview

A typical geospatial query can be divided and pipelined into several sub-phases. In general, these are (1) parsing, (2) indexing/filtering, and (3) refining. In the first phase, input data is usually converted from textual or binary format to in-memory spatial data structures. These spatial data structures are then used to build spatial index on input datasets. Using the indexes, spatial data objects from the input are filtered to keep only operation relevant objects in memory. Finally, the compute intensive geospatial computation is applied only to relevant filtered spatial objects to produce query results. In this section, we explore these phases for spatial cross-matching operation in detail and explain how can they be adapted for the proposed CPU/GPU hybrid architecture.

**Figure 1:**
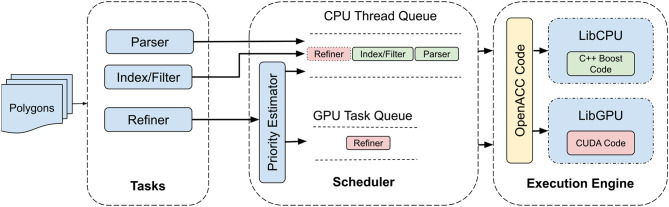
Hybrid CPU/GPU architecture to accelerate geospatial query processing.

There are three major logical components of our system; tasks, scheduler and execution engine. Figure 1 illustrates the interaction among these components when deployed over a single node.

### 3.1. Query Tasks

After reading input spatial data, the system divides the cross-matching workflow into pipeline of tasks. This pipelining is essential since it allows different stages of the query operation to be scheduled and executed on different processing units available on the underlying compute node. In this particular case, these processing units include a multi-core CPU and a GPGPU. The decision to execute a particular task on a processing unit is dependent on the characteristics of that task. For instance, parsing, indexing, and filtering tasks although can be parallelized but have processing dependencies which make them unsuitable to be executed on GPU. The refiner task, however, allows for computation at pixel level and thus can easily be parallelized on GPU cores.

### 3.2. Scheduler

The hybrid architecture allows for the system to fully exploit all of the processing units available in a modern compute nodes. As discussed earlier, a workload is divided into parse, index/filter and refine tasks. Considering available parallelism, the first two tasks are always executed on a multi-core CPU whereas the refiner task, ideally is processed on the GPU. However, despite being suitable for GPU parallelism, running a task on GPU have overheads that need to be justified by the actual processing. For instance, data needs to be copied from main memory to GPU memory, which is an extra step in case a particular task needs to be executed on the GPU. This data copying cost can easily surpass the benefits of accelerating the task processing through GPU. In such cases, it would rather make more sense to be content with relatively limited parallelism provided by a multi-core CPU which can still outperform the GPU in terms of overall processing time.

The second important component in our system is the scheduler. As illustrated in Figure 1, the scheduler has two sets of queues; namely task and thread queues. The scheduler assigns new incoming tasks to available workers using either first come first server (FCFS) or priority queue (PQ). For FCFS, parse and index/filter tasks are always assigned to the CPU threads whereas the refiner task is either executed on CPU or GPU based on its position in task queue and the availability of the processing unit. Alternatively, for PQ, the scheduler assigns a priority to the incoming tasks based on their potential speedup gain if executed on GPU. Higher the priority, higher is the chance that the task will be executed on GPU. Note here that parse and index/filter tasks are always assigned the lowest priority so that they must always execute on CPU workers.

### 3.3. Execution Engine

Finally, the execution engine consists of libraries for actual spatial computation. In particular, the engine comprises of two separate libraries to perform the refinement task on CPU and GPU.

#### 3.3.1. CPU Refiner

We employ the Boost library to conduct refinement of polygonal pairs on CPU. The boost library conducts the computation in a serial manner, that is, in each tile, the polygon set is processed on CPU thread one after another. Then multiple tiles are executed in parallel on multiple CPU threads.

#### 3.3.2. GPU Refiner

We extend the PixelBox algorithm to conduct refinement of polygonal pairs on GPU. First, the information of two polygon sets is copied from CPU memory to GPU memory. Each polygon pair in a tile is scheduled to a thread block. The MBB of each polygon pair is partitioned to individual pixels, and the relative position of each pixel is evaluated against the two polygons. Then the number of pixels within each of the polygons is used as a measure of intersection and union area. Finally, the results are copied back to CPU to evaluate the accuracy of the computation.

## 4. Cross-Matching Query Workflow

The cross-matching query workflow can broadly be categorized into three phases; (1) parse, (2) filter, and (3) refine. In this section, we explain these phases in detail with respect to their implementation on the proposed hybrid architecture. Algorithm 1 presents a logical flow of the cross-matching query. Given two datasets, first, they are loaded from source into memory. Since both datasets are independent of each other at this stage, they can be loaded simultaneously in different CPU threads. The load process also partitions both of the datasets into tiles according to the user-selected *partitionMethod*. Once partitioned and loaded in memory, each pair of tiles from the two datasets is passed on to the index/filter stage to produce a set of partial results. Finally, these results are refined using either CPU or GPU refiner considering scheduling heuristics.

**Algorithm 1:** Cross-Matching Query

1: procedure Query(*dataset*_1_, *dataset*_2_, partitionMethod)
2: /* executed in parallel */
3: *tiles*_1_ = *Load*(*dataset*_1_, *partitionMethod*)
4: *tiles*_2_ = *Load*(*dataset*_2_, *partitionMethod*)
5: **for** each pair (*tile*_1_, *tile*_2_) in (*tiles*_1_, *tiles*_2_) **do**
6: *partialResults* = *IndexFilter*(*tile*_1_, *tile*_2_)
7: *Refiner*(*partialResults*)
8: **end for**
9: **end procedure**

### 4.1. Input Data Parsing

The steps in the parsing phase are listed in Algorithm 2. Input geospatial data in our case consists of polygons and is represented either in textual or binary format. Once loaded into memory, the data is partitioned according to user-specified partitioning method. The details of spatial partitioning are beyond the scope of this paper. However, for practical purposes, we assume the partition to be *Fixed Grid* which divides the input data space in tiles of equal dimensions. After partitioning each tile is then processed further to compute tile specific spatial parameters. Furthermore, for each polygon in the tile number of vertices, MBB and list of coordinate vertices are computed which then are aggregated to derive tile specific parameters. One important observation to note here is that the parsing phase is not optimized for GPU processing since processing is mostly done at the object level. Therefore, the opportunity for parallelism is fairly limited. Due to the this, parsing phase is always scheduled to be executed on CPU in parallel on multiple available cores.

**Algorithm 2:** Parse Phase

1: **procedure** Prepare(tile)
2: Initialize *tileConfig*
3: /* executed in parallel */
4: **for** each spatialObject in tile **do**
5: *vCount* = *countVertices*(*spatialObject*)
6: *mbb* = *extractMBB*(*spatialObject*)
7: List *coord* = *extractCoordinates*(*spatialObject*)
8: *tileConfig*.*addObject*(*mbb, vCount, coord*)
9: **end for**
10: return *tileConfig*
11: **end procedure**
12: **procedure** Load(dataset, partitionMethod)
13: Read textual spatial data in memory
14: Initialize List *tileConfigs*
15: List *tiles* = *partition*(*dataset, partitionMethod*)
16: **for** each tile in tiles **do**
17: *tileConfigs*.*add*(*Prepare*(*tile*))
18: **end for**
19: return *tileConfigs*
20: **end procedure**

### 4.2. Indexing/Filtering

Algorithm 3 lists the pseudo-code for the second phase of the geospatial cross-matching operation. In order to accelerate query processing, a local index is created using MBBs of polygons from every pair of tiles. In our implementation, we created Hilbert R-Tree index. The index is then used to filter out polygons whose MBBs intersect with each other. It is worth noting that although this process consists of geospatial computation, the complexity of it is relatively simpler since the intersection is computed for regular bounding boxes only and not for actual polygons. Consequently, the results returned by this phase are regarded as partial results containing relevant geospatial objects only. Again, since this stage also operates at object level, it is better suited to be parallelized on multiple CPU cores only.

**Algorithm 3:** Index/Filter Phase

1: **procedure** IndexFilter(*tile*_1_, *tile*_2_)
2: /* create local Hilbert R-Tree index */
3: *localIndex* = *tile*_2_.*createIndex*()
4: Initialize List *results*
5: /* filter spatial objects whose MBBs intersect */
6: **for** each *object*_1_ in *tile*_1_ **do**
7: /* use index to get qualifying objects */
8: **for** each qualifying *object*_2_ from *tile*_2_ **do**
9: **if** MBBs of *object*_1_ and *object*_2_ intersect **then**
10: append both to *results*
11: **end if**
12: **end for**
13: **end for**
14: return *results*
15: **end procedure**

### 4.3. Result Refinement

The final phase in the cross-matching operation is to refine the partial results generated in the previous step (section 4.2) and produce polygons that actually intersect. In addition to just returning intersecting polygons, this phase also computes the area of intersection of pairs of intersecting polygons as noted in Algorithm 4. The scheduler discussed in section 3.2, estimates parameters, such as the number of polygons, geospatial data size in partial results and data movement cost from CPU main memory to GPU memory. Based on these parameters, it comes up with a priority value assigned to the incoming task which is then used to determine whether to execute the refiner phase on CPU cores or on GPU. In case the priority values are low based on speedup estimations, the refiner task is simply executed linearly on CPU for each pair of polygons from partial results set. However, if the potential estimated speedup is considerable, the refiner task is executed on GPU using an extension of the PixelBox algorithm (Wang et al., 2012).

**Algorithm 4:** Refiner Phase

1: procedure CPURefiner(partialResults)
2: Initialize List *results*
3: **for** each pair (*obj*_1_,*obj*_2_) in *partialResults* **do**
4: **if** *obj*_1_.intersects(*obj*_2_) **then**
5: *results*.*append*(*obj*_1_, *obj*_2_)
6: **end if**
7: **end for**
8: return *results*
9: **end procedure**
10: **procedure** GPURefiner(partialResults)
11: *copyFromCpuToGpuMem*(*partialResults*)
12: /* executed in parallel separate thread blocks */
13: **for** each pair (*obj*_1_,*obj*_2_) in *partialResults* **do**
14: *MBB* partitioned to each individual pixel
15: Relative position of each pixel w.r.t (*obj*_1_,*obj*_2_)
16: *results* = *Numberofpixelsineachobj*
17: **end for**
18: *copyFromGpuToCpuMem*(*results*)
19: **end procedure**
20: **procedure** Refiner(partialResults)
21: /* estimate parameters for scheduling */
22: *params* = *estimateParameters*(*partialResults*)
23: *executor* = *evaluateExecutor*(*params*)
24: **if** executor == CPU **then**
25: return *CPURfiner*(*partialResults*)
26: **else**
27: return *GPURfiner*(*partialResults*)
28: **end if**
29: **end procedure**

## 5. Generalized Spatial Cross-Matching Algorithm

### 5.1. PixelBox Algorithm

PixelBox is a GPU algorithm that computes areas of intersection and union for a set of input polygon pairs. The algorithm first pixelizes the minimum bounding box of each polygon pair and utilizes ray casting method to determine every pixel's position relative to input polygons. Since computation at every pixel is independent of each other, the method is very well-suited to be parallelized on GPU threads. However, this also means that the computation complexity of the algorithm increases dramatically with higher resolution images having more number of pixels. To reduce this complexity and make the algorithm more scalable, PixelBox combines the *pixelization* approach with *sampling box* method. Sampling Box exploits the spatial locality characteristics of pixels to compute areas of polygon intersection and union region by region instead of pixel by pixel. This allows the PixelBox algorithm to perform much more efficiently and improves overall scalability.

The original PixelBox algorithm was designed to operate specifically on spatial objects identified by medical analytics in pathological image datasets (Wang et al., 2012). Polygons represented in such datasets were assumed to have edges along horizontal/vertical directions only. Additionally, the algorithm was designed to handle polygons with vertices represented in integer-based coordinates only. Consequently, the PixelBox algorithm could not be applied directly to real-world datasets containing polygons having edges with any orientation and vertices represented by floating-point numbers.

### 5.2. Geo-PixelBox Workflow

**Figure 2:**
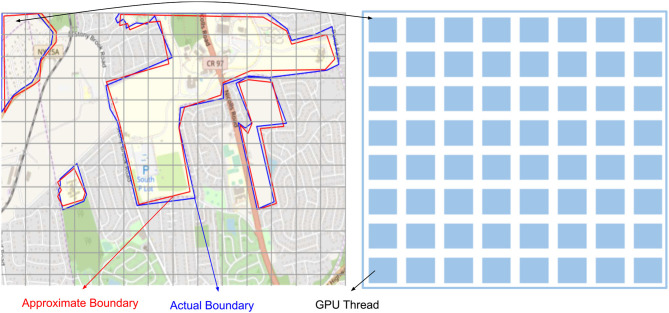
Approximation of geospatial object with a scaling factor *k* to convert vector based expression to pixel based expression.

In order to generalize the PixelBox algorithm for geospatial datasets, we created a multi-step workflow termed as *Geo-PixelBox*. Before processing the data on GPU, we converted the original geospatial polygon datasets to integer-valued vertex coordinates. This is achieved by multiplying the original minimum bounding rectangle of each polygon pair by a scaling factor *K*, which is varied depending on the floating point-valued area of the original MBB. Figure 2 illustrates this approximation process. This allows us to compute and compare the intersection and union areas on GPU. However, it must be noted that there is a loss of accuracy during the data conversion process. For each polygon pair, we created a set of values for the factor *K* and keep *K* as small as possible while maintaining the error of computation within 5%.

The left, right, bottom and top boundaries of a minimum bounding rectangle are used to compute the original MBB area. A new boundary is formed by rounding down the lower boundaries and rounding up the upper boundaries by multiplying the factor *K*. Assume each pixel occupies unit area, then the number of pixels within the new boundary accounts for the polygon area after scaling. An error of conversion is evaluated between the original area and the new area. We set a threshold of error as 0.05 and compute the error using formula

(1) $$\begin{array}{l} error=\frac{abs\left(\frac{area_{scaling}}{K^{2}}-area_{original}\right)}{area_{original}} \end{array}$$

For qualified polygon pairs, we determine the relative position of each pixel at location (x,y) with the two polygonal pairs, using formula (for an upward edge)

(2) $$\begin{array}{l} x\leq xs\left[i\right]+\left(y-ys\left[i\right]\right).\frac{xs\left[i+1\right]-xs\left[i\right]}{ys\left[i+1\right]-ys\left[i\right]} \end{array}$$

(3) $$\begin{array}{l} y\geq ys\left[i\right],y\leq ys\left[i+1\right] \end{array}$$

where (xs [i], ys [i]) and (xs [i+1], ys [i+1]) are two vertices of each edge of a polygon. Finally, determine the area of intersection and union by counting number of pixels falling into each of these two polygons.

### 5.3. Implementation

#### 5.3.1. Geometry Based Implementation

Most of the available geospatial processing libraries operate on geometries of objects since they primarily utilize CPU and its comparatively limited parallelism. We used *Geometry Engine Open Source* (GEOS) (OSGeo, 2019) library which provides implementations of fundamental geospatial algorithms conforming to *Open Geospatial Consortium* (OGC, 2019). In particular for spatial cross matching, GEOS usually issues repeated Point-in-Polygon queries against the fixed query polygon to generate results satisfying *intersects* and *contains* predicates. GEOS utilizes implementations of *Fast Point-in-Polygon* along with spatial indexes, such as *QuadTree* and *STRtree* to optimize the query performance.

#### 5.3.2. Pixel Based CUDA Implementation

PixelBox (Wang et al., 2012) provides a sophisticated native CUDA based implementation designed to operate on Nvidia GPUs. We extend the same algorithm with modifications discussed in section 5.2. We first scale MBRs of query polygons followed by pixelizing the polygon space comprised of combined Minimum Bounding Rectangle of query polygons. Pixelization allows for determining each pixel's relative position totally independent of each other. Then using sampling boxes technique, we reduce the processing space and thus the total compute intensity. Finally, we combine the pixelization and sampling box approaches to efficiently compute cross-matching union and area of intersection of query polygons.

#### 5.3.3. Pixel Based OpenACC Implementation

Finally, we implemented the Geo-PixelBox in c++ primarily to experiment with OpenACC. However, since OpenACC is more portable than CUDA, switching on/off a single parameter i.e., *-acc* to the PGI compiler, we can have the same piece of code running on CPU and GPU.

We implemented the same pixel-based algorithm, described in section 5.3.2, in C++. In essence, all pixels of minimum bounding rectangle were compared against the boundaries of intersecting polygon. Then we applied OpenACC compiler directives to accelerate the code for GPU execution. In particular, for OpenACC accelerated GPU execution, there were two levels of parallelism; first on polygon pairs level achieved by multiple gangs, and second is on polygon pixel level achieved by vectors.

## 6. Experimental Evaluation

### 6.1. Experimental Setup

We used a single node having access to a multi-core CPU and two GPUs. The multi-core CPU was able to support up to 16 parallel threads. The node had GeForce GTX 750 and Tesla K80 GPUs. The former GPU had 640 cores, 5 streaming multiprocessors (SM), 2 GB GDDR 5 memory with 86.4 GB/s memory bandwidth, 256 KB register per SM, and 64 KB shared memory per SM. On the other hand, Tesla K80 had 4,992 cores, 26 SMs, 24 GB GDDR5 memory with 480 GB/s memory bandwidth. At the time of writing, the price for GTX 750 is $150 and K80 is $5,000.

In order to compile pixel based c++ code accelerated by openacc, we used PGI compiler version 18.10-1 on a 64-bit target machine running x86-64 CentOS. We configured openacc with 32 gangs, 1 worker and vector length of 1,024. All of the experimental details are summarized in Table 1.

**Table 1:** Summary of experimental setup.

	**GTX 750**	**Tesla K80**	**OpenACC Configuration**	
# of cores	640	4,992	# of gangs	32
Streaming multiprocessors	5	26	# of workers	1
Memory (GDDR5)	2GB	24GB	Vector length	1,024
Memory bandwidth (GB/s)	86.4	480	Compiler	PGI
Cost ($)	150	5,000	PGI version	pgc++ 18.10-1 64-bit

### 6.2. Experiment Design

#### 6.2.1. Dataset

We applied the pixelization method to geospatial vector dataset from Natural Earth Data (Natural Earth, 2018) in ESRI shapefile format with UTF-8 character encoding. We used two datasets in shapefile format containing polygon representation of U.S.A state boundaries and urban areas in the United States. Each of these files contained vector data at the scale of 1:10 million with 1 inch representing 158 miles. The data was replicated multiple times to stress test our system with a larger number of intersecting polygons.

#### 6.2.2. Scaling Factor and Error Rate

To find the optimal scaling factor, we varied the scaling factor *k* from the set which ends up with a different number of pixels. We multiplied the coordinates in original input data by the estimated scaling factor *k* to achieve the integer representation of geospatial polygon sets. The selection of *k* depends on the area of each polygon. Furthermore, the value of *k* determines the number of pixels after approximation, which is directly correlated to GPU performance. In our experiments, *k*'s value was set to 50, 100, and 150, respectively.

In order to study the effect of scaling factor on error rate, we selected three; smallest, medium and largest, represented polygons from our dataset. The areas of these polygons were computed in *inches*^2^ using latitude and longitudes of polygon vertices. These polygons with areas (a) 0.012631360, (b) 0.050525440, (c) 0.2021017588 were used to derive the relationship between error vs. scaling factor, and the number of pixels vs. scaling factor. 9 decimal digits are considered here for the coordinates of vertices. Using the natural earth data as input, we investigated the relationship of accuracy and number of pixels. With up to 5% error in area computation, less number of pixels is desired.

#### 6.2.3. Refinement Task Implementations

We evaluated several implementations of the refinement task on CPU and GPU. For CPU, the default implementation was based on boost and geos library performing geometry based cross-matching task. All of the following evaluation results mentioning CPU refer to this default version unless noted otherwise. Since OpenACC allowed accelerating C++ code for GPU, we implemented Geo-PixelBox in C++ as well. The same code was executed on CPU when appropriate compiler directives were disabled. This version is referred to as *Geo-PixelBox-CPU* throughout the evaluation.

#### 6.2.4. GPU Accelerated Implementations

For GTX750 and K80 GPUs, we used two implementations of Geo-PixelBox algorithm, i.e., Geo-PixelBox implemented in C++ and accelerated for GPUs using OpenACC and Geo-PixelBox implemented in CUDA. During our evaluation, these are referred to as *Geo-PixelBox-OpenACC* and *GeoPixelBox-CUDA*. Table 2 summarizes these terminologies for reference. The IO time was excluded from timing results unless noted otherwise. For both of the GPUs, the number of block was selected as $\frac{N}{10}+1$, where *N* is the number of polygon pairs to be processed. Similarly, the number of threads per block was selected as 1,024 for both GPUs.

**Table 2:** Summary of terminologies used in evaluation.

**Implementation**	**Description**
CPU	C++ geometry based implementation
Geo-PixelBox-CPU	C++ pixel based implementation
Geo-PixelBox-OpenACC	C++ pixel based implementation accelerated by OpenACC
GPU, Geo-PixelBox-CUDA	CUDA pixel based implementation

In order to evaluate the performance and efficiency of CPU-GPU hybrid architecture, we employed several combinations of processing units. For a baseline comparison, we used a single-threaded CPU to execute all the cross-matching operation tasks. We then compared the performance of different combinations of 1GPU, 1CPU-GPU, 4CPU-GPU, and 16CPU-GPU approaches for several datasets. These datasets mostly consisted of polygon pairs within a single tile ranging from 10,000 to 280,00 pairs. To study the performance, we computed speedup as a ratio between *i* number of CPU threads plus GPU and *i* number of CPU threads, where *i* = 14 and 6

(4) $$\begin{array}{l} Speedup=\frac{T_{iCPU}}{T_{iCPU+GPU}} \end{array}$$

One important consideration resulting from partitioning is data skew which is inherent to geospatial datasets. Having data skew, the performance of the overall system is only as good as the slowest worker having most of the data. This is a well-studied field in literature and thus is beyond the scope of this study. In order to mitigate the effects of data skew among partitioned tiles, we generated synthetic data by duplicating the tile with 280,000 polygon pairs. These number of tiles were varied from 20, 50, 100, 200 to 400 tiles for different experiments.

Finally, we also measured the effects of two scheduling methods; First Come First Server (FCFS) and Priority Queue (PQ), in terms of speedup ratio.

### 6.3. GPU Cost Effectiveness

**Figure 3:**
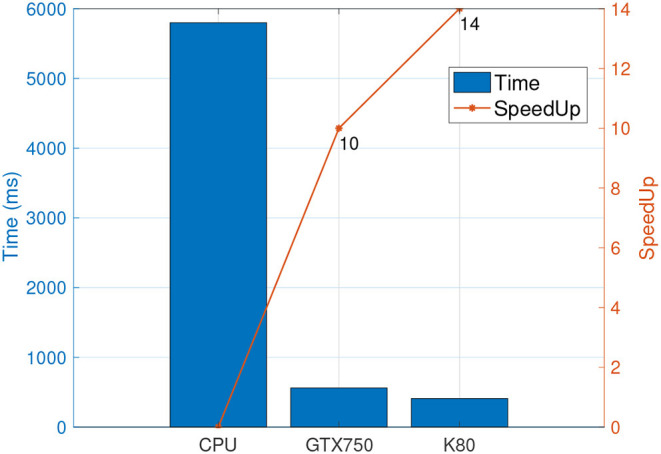
Time spent in refinement phase by GPUs and a single threaded CPU along with their respective speedup.

After input data was converted into pixel representation, we employed two GPUs to compute the time of refinement of a single tile and running time of the same input data on CPU using boost library (Figure 3). The CPU running time was 5,799 ms. For the same refinement task GPUs GTX750 and K80 took 561 and 401 ms, respectively. The corresponding speedup ratio was 10× for GTX 750, and 14× for K80.

As noted earlier, the cost of GTX750 is ~$150 whereas K80 costs around $5,000. This means that we achieved 1.4× speedup while paying about 33× more. Therefore, we may safely conclude that GTX750 is much more cost effective than K80 for the dataset and geospatial operation in consideration.

### 6.4. CPU vs. GPU Speedup Baseline

**Figure 4:**
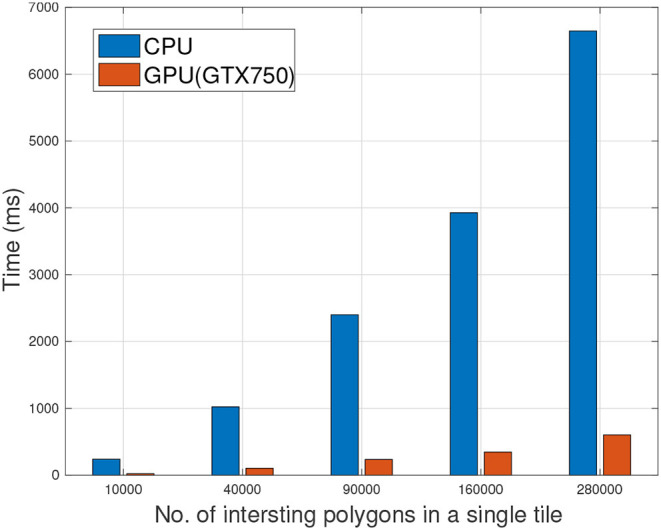
Comparison of refinement phase executions on single threaded CPU vs. Hybrid (1CPU-GPU) with respect to increasing number of polygons, i.e., spatial computation.

In order to come up with a baseline for our evaluation, we compared the running times of refinement task on a CPU with single thread vs. running time of the same task on GPU. Figure 4 shows this execution time comparison of refinement task for increasing dataset size. As the number of intersecting polygons increased from 10,000 to 280,000, the time of refinement on a single CPU with 1-thread increased from 239 to 6,647 ms. Alternatively, for the same operation on the same dataset for GTX750 the time increased from 22 to 602 ms. Overall the speedup ratio varied between 10× to 11× for different number of intersecting polygons in a single tile.

### 6.5. Geo-PixelBox Scaling Accuracy

As stated in section 5, we have to convert geospatial polygons with floating point representation into integer representation to use the PixelBox algorithm for GPU enabled geospatial cross-matching. We tested the accuracy of approximation to enable GPU-based geospatial cross-matching operation. The error of approximation and resulting number of pixels for a geospatial polygon were evaluated with respect to different scaling factors *k*.

**Figure 5:**
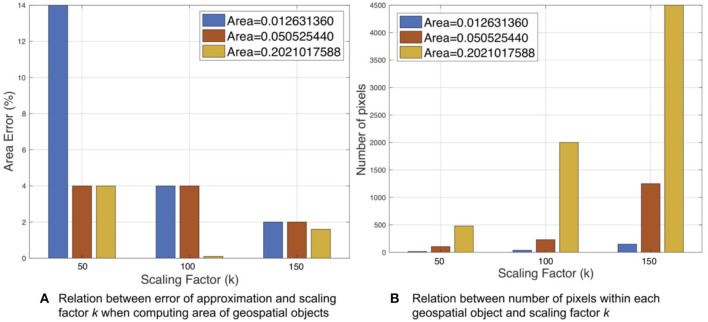
Accuracy of approximation to enable GPU based cross matching operation with respect to scaling factor *k*.

The polygon size was represented by its area *A*_*p*_ computed based on map coordinates. A polygon size on the order of *A*_*p*_ ≈ 0.01 has a 14% error after approximating to integer-based coordinates with scaling factor *k* = 50 (Figure 5). The scaling error was continuously reduced to <2% when *k* was increased to 150. A relatively larger polygon with area *A*_*p*_ ≈ 0.05 can achieve 4% error with *k* = 50. The largest polygon where *A*_*p*_ ≈ 0.2 shows a similar result to *A*_*p*_ ≈ 0.05. In addition, the number of pixels vary with respect to different scaling factor *k*. The polygon with *A*_*p*_ ≈ 0.01 has <500 pixels when *k* = 150. However, under same *k*, the larger polygons *A*_*p*_ ≈ 0.05 results in 1,500 pixels and *A*_*p*_ ≈ 0.2 results in 4,500 pixels.

### 6.6. Geometry vs. Pixel Based Cross Matching on CPU

**Figure 6:**
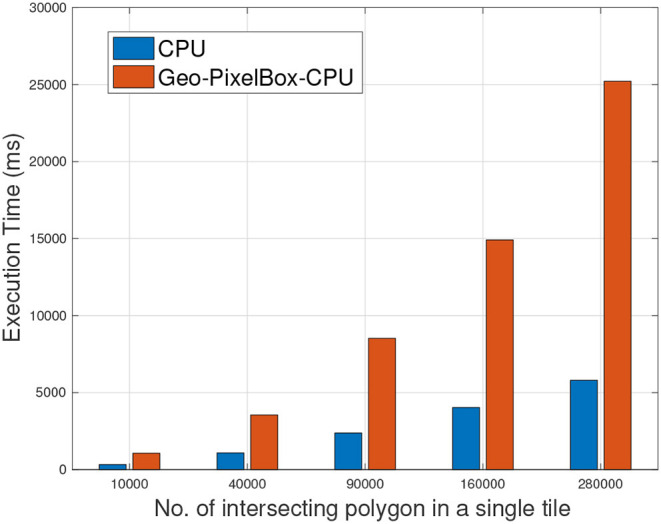
Geometry based cross matching vs. Pixel based cross matching operation when executed on CPU.

In section 6.4, we've discussed the baseline performance of geometry based CPU vs. pixel based CUDA implementation on GPU. To extend this further, we now analyze the performance of pixel based cross-matching algorithms on CPU as well as when accelerated by OpenACC. Figure 6, illustrates the results affirming the hypothesis that pixel based method is not suitable to be parallelized by CPU. For all experiment datasets, geometry based implementation always outperformed pixel based implementation when cross-matching operation was executed on CPU.

### 6.7. Pixel Based Cross Matching on CPU vs. GPU

**Figure 7:**
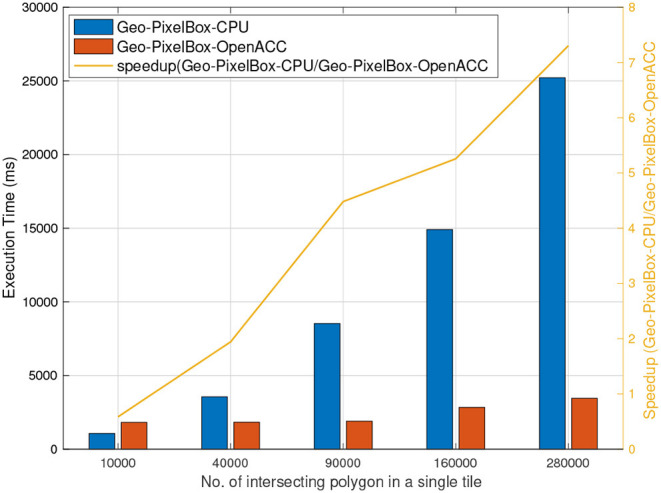
Pixel based cross matching executed on CPU vs. on GPU accelerated by OpenACC.

Figure 7 shows the execution time and speedup when Geo-PixelBox was executed on CPU against when it was accelerated using OpenACC to be executed on GPU. These results again support the fact that the performance of pixel based cross-matching is proportional to the availability of parallelism in the processing unit.

### 6.8. Geometry Based Cross Matching on CPU vs. OpenACC Accelerated Pixel Based Cross Matching on GPU

Results from sections 6.6 and 6.7 clearly establish the fact that pixel based cross-matching is a much better fit to be parallelized on GPU as compared to CPU having limited parallelism. However, it must be noted, that geometry based cross-matching when executed on CPU does have considerable performance results. While results from section 6.4 unarguably show pixel based cross-matching implemented in CUDA to clearly outperform geometry based cross-matching, in this section we evaluate the performance of OpenACC accelerated pixel based cross-matching.

**Figure 8:**
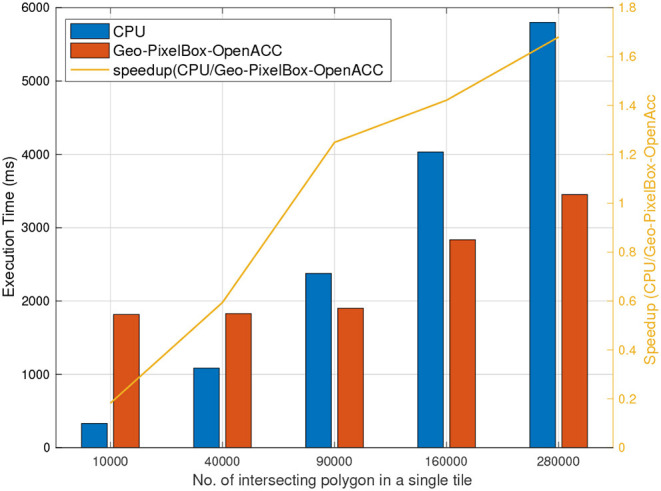
Geometry based cross matching executed on CPU vs. Pixel based cross matching on GPU accelerated by OpenACC.

Figure 8 shows that accelerating pixel based cross-matching with OpenACC does not yield as high speedup as compared to CUDA. In fact, for smaller datasets, geometry based cross-matching outperforms pixel based OpenACC accelerated GPU cross-matching. Although, this can very well be associated with IO overheads as discussed in section 6.9, it can safely be assumed that for geospatial datasets in particular, and in general GPU code implemented in CUDA gives more performance gain than GPU code accelerated with OpenACC.

### 6.9. Data Copying Overheads for GPU Computations

As mentioned in section 3.2, the cost of copying data from main memory to GPU memory can dominate the total processing time. This can reduce or in some cases surpass the benefits achieved from accelerating the task through GPU. To study the effects of data copying cost we compared the percentage time spent in IO by a single CPU thread and GPU for the refinement task.

**Figure 9:**
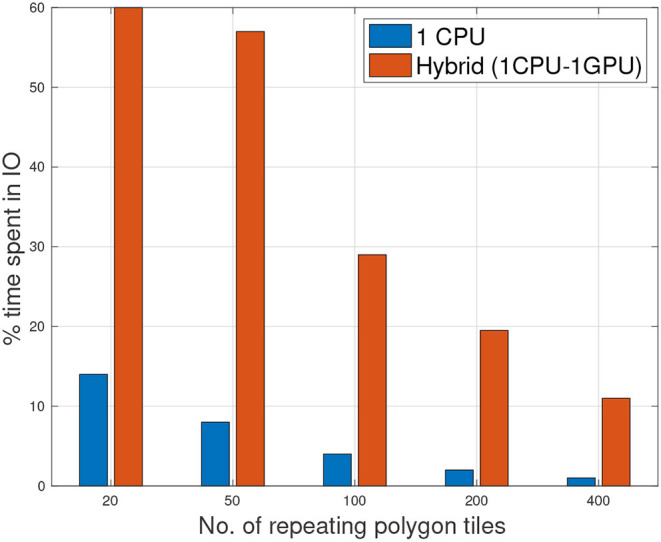
Percentage of time spent in IO of 1CPU/GPU and single threaded CPU with increasing number of polygon tiles.

In our experiments, the total time to perform refinement task using 1CPU and 1CPU-1GPU was almost similar ranging from 0.8 to 1 s. However, although CPU-GPU was able to accelerate the processing and cut on the computation, it spent considerably more time in IO as compared to CPU. Figure 9 shows the ratio of IO vs. total running time. By using 1 CPU, the IO took 14% of the total time when there were 20 tiles to process. This value reduced to <1% when 400 tiles were processed. However, for CPU-GPU, the IO/total ratio was 60% when there were only 20 tiles. This value reduced to 11% with 400 tiles were processed. This comparison clearly illustrates that careful consideration is needed in terms of dataset size and potential speedup when accelerating jobs on GPU.

### 6.10. CPU-GPU Hybrid Geospatial Cross-Matching

**Figure 10:**
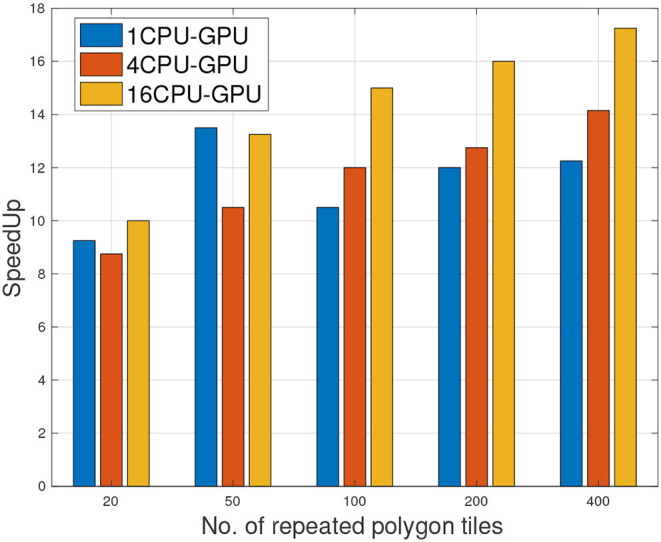
Speedup of 1CPU/GPU, 4CPU/GPU, 16 CPU/GPU with respect to single threaded CPU with increasing number of polygon tiles.

Benchmarking GPU accelerated tasks with a single threaded CPU is not fair by any means. Modern CPUs are very capable of parallelizing tasks although relatively at a limited scale. For a fair comparison, we extended our 1CPU-1GPU hybrid approach to 4CPU-GPU and 16CPU-GPU. Figure 10 show the effects of adding more CPUs to the computation. Since parsing and index/filtering tasks are always scheduled on CPU, more CPUs meant more workers thus further accelerating these stages. In conjunction with the refiner task running on GPU, the maximum speedup achieved was 17× when 400 tiles were processed using 16CPU-GPU.

### 6.11. Scheduling Methods

**Figure 11:**
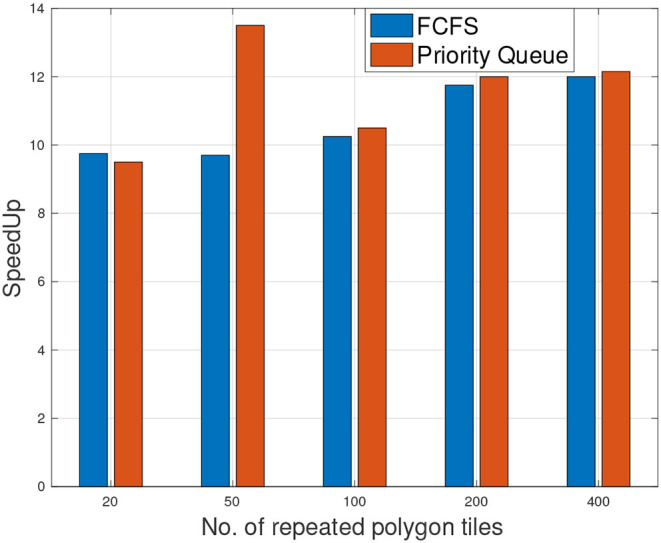
Speedup of 1CPU/GPU under two scheduling methods (Priority Queue and FCFS) with respect to a single threaded CPU with number of polygon tiles.

Figure 11 shows the speedup of hybrid 1CPU-GPU with respect to a single threaded CPU for two different scheduling methods. Interestingly, the results demonstrate that both PQ and FCFS scheduling methods achieve similar speedup ratios when the number of tiles increased from 20 to 400.

## 7. Conclusions

Spatial data analysis is inherently compute intensive making it a good candidate for acceleration via parallel processing units, such as GPU (Wang et al., 2015). Recent works in this domain (Zhang et al., 2015a; Liang et al., 2016) have tried addressing this problem but are mainly limited to medical datasets for health care analytics. In this study, we extend our proposed CPU-GPU hybrid approach (Gao et al., 2018) to perform spatial cross-matching on geospatial datasets focusing on potability, high resource utilization, and performance.

Our proposed workflow creates a pipeline of subtasks for cross-matching operation. These subtasks are then dynamically scheduled on CPU or GPU and are able to utilize parallelism at both inter- as well as intra-object level. By distributing multiple polygon pairs to different thread blocks, we were able to achieve object level parallelism. At the same time, intra-object parallelism was achieved by distributing pixels onto multiple GPU threads. We extensively studied the effects of various systems parameters including data size, speedup, approximation error, and cost effectiveness.

By implementing *Geo-PixelBox* algorithm in CUDA as well as in OpenACC accelerated C++, we studied the trade-off between performance and portability for GPU implementations. While CUDA based implementation achieved the best performance in terms of job execution time, OpenACC accelerated C++ code was definitely more portable and extendable. In particular for cross-matching operation, the former was able to achieve up to 14× speedup whereas the later achieved about 2× speedup compared to CPU based implementation.

In terms of the dataset, we studied the effects of data copying overheads from main memory to GPU memory when parallelizing geospatial cross-matching operation over GPU. As expected, our experiments indicate that the high ratio of IO vs. computation for CPU-GPU approach should be mitigated by increasing dataset size to achieve better performance. Similarly, our results indicated that the number of polygon pairs within a single tile does not affect the speedup ratio. As more polygon pairs exist in a single tile, the computation time for both CPU and GPU was increased and a similar speedup ratio was observed.

Finally, we studied the performance of our system when proposed pipeline was executed on combined multi-threaded CPU and GPU. Our results show that a similar speedup ratio was achieved when a combination of CPU threads and GPU were employed. This is because GPU is more efficient to perform refinement operation (10× speedup), and therefore can take more tasks than CPU threads. This fact was further confirmed by the scheduling methods, where the priority queue and First-Come-First-Serve demonstrated similar results.

## Data Availability Statement

The datasets generated for this study are available on request to the corresponding author.

## Author Contributions

FB and FW contributed to the major design and conception of the work. JK provided the initial data set and insights for the project. CG implemented and carried out experiments and provided interpretation and analysis for the work along with FB and DT. FB wrote the manuscript with support from CG and DT. DT contribution helped in improving overall intellectual value of the work. FB, CG, and DT approved the final manuscript under FW supervision.

## Conflict of Interest

The authors declare that the research was conducted in the absence of any commercial or financial relationships that could be construed as a potential conflict of interest.
